# Hydrophobic Diffusion Media via Electrografted Organosilicons Enable Competitive Performance in Polymer Electrolyte Fuel Cells

**DOI:** 10.1021/acselectrochem.5c00333

**Published:** 2025-11-04

**Authors:** Irene Sinisgalli, Adrian Mularczyk, Antoni Forner-Cuenca

**Affiliations:** Electrochemical Materials and Systems, Department of Chemical Engineering and Chemistry, 3169 Eindhoven University of Technology, P.O. Box 513, 5600 MB Eindhoven, The Netherlands

**Keywords:** fuel cells, hydrophobicity, organosilicons, diazonium, grafting, gas diffusion electrodes

## Abstract

Hydrophobic surface modification of porous carbon materials is critical for the performance and durability of polymer electrolyte membrane fuel cells and many other electrochemical technologies. However, conventional treatments rely on dip coating approaches using polymer dispersions containing fluorinated compounds (i.e., polytetrafluoroethylene), which pose environmental hazards and yield heterogeneous surfaces with limited control over wetting behavior and poor durability. Here, we introduce a facile method that exploits the facile electroreduction of 4-nitrobenzodiazonium tetrafluoroborate to generate aryl radicals and initiate a radical chain reaction that enables the grafting of vinyl-terminated organosilicon compounds. This method proceeds under ambient conditions and at milder potentials than traditional vinylic electrografting. We investigate three organosilicon derivatives—allyltriisopropylsilane, acryloxymethyl­trimethylsilane, and monomethacryl­oxypropyl-terminated polydimethylsiloxane—as electrografted coatings to tailor surface wettability. We perform microscopic, spectroscopic, and contact angle measurements and electrochemical characterization to correlate chemical moieties with the resulting wettability and electrochemical performance in fuel cells. We find that the electrografted coatings form a covalently bonded, thin layer that significantly reduces the solid surface energy of the carbonaceous substrate, reaching values close to those of polytetrafluoroethylene. Additionally, we find a correlation between surface energy and fuel cell performance, where the less hydrophobic coatings show cell flooding under more humid conditions. Polydimethylsiloxane-based coating outperforms the commercial baseline (polytetrafluoroethylene) in operando fuel cells, which paves a promising pathway for this class of materials. Importantly, this study highlights the potential of fluorine-free alternatives to traditional fluorinated hydrophobic treatments, offering a more sustainable and environmentally friendly approach without compromising performance.

## Graphical abstract

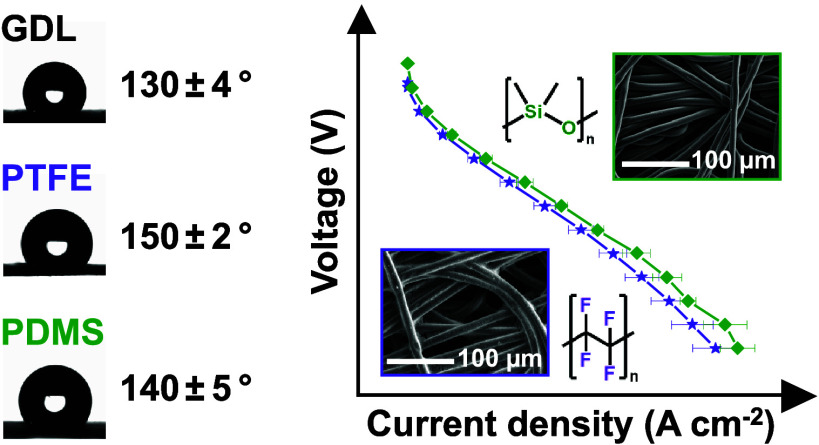

## Introduction

The development of (super)­hydrophobic surfaces is pivotal in various industries, ranging from energy conversion technologies^1^ to textiles^2^ and biomedical devices.^3^ Fluorinated polymers, such as polytetrafluoroethylene (PTFE), have been the materials of choice due to their exceptional water repellency, chemical stability, and thermal and mechanical resilience.^4,5^ However, the use of certain fluorinated compounds, such as per- and polyfluoroalkyl substances (PFAS), may be restricted in the next years due to growing evidence of their environmental accumulation^6,7^ and its demonstrated adverse health effects.^8^ While PTFE itself is considered less hazardous than many other PFAS, largely because of its high stability and resistance to degradation under mild conditions, it remains of paramount importance to develop sustainable alternatives that offer similar properties while safeguarding the environment.^9,10^ In fact, PTFE production often relies on harmful PFAS-based chemical precursors, such as perfluorooctanoic acid.^11^ Among the various molecular candidates, organosilicon-based materials (with their tunable surface chemistry and hydrophobicity) have emerged as promising contenders.^12,13^ Here we explore the application of organosilicon-based grafted layers to tackle a pressing challenge in electrochemical energy conversion and storage technologies.

Electrochemical energy technologies (e.g., fuel cells, batteries, electrolyzers) heavily rely on the use of fluoropolymers to impart hydrophobicity or proton conductivity,^14^ and with the rising adoption of renewable energy sources, the demand for electrochemical systems is expected to significantly grow in the coming years.^15^ In polymer electrolyte membrane fuel cells (PEMFCs), effective water management is critical for maintaining optimal performance and preventing flooding.^16^ The gas diffusion layer (GDL), a porous carbonaceous substrate, facilitates the transport of reactant gases and the removal of water produced at the cathode.^17^ Traditionally, PTFE dip-coating is used to impart hydrophobicity to the GDL (Figure 1a).^18^ And while functional, this method results in heterogeneous coatings^19^ and weak physical adhesion to the substrate, leading to polymer detachment, hydrophobicity loss, and performance decay over time.^20^ To overcome these limitations, researchers have explored alternative methods such as CF_4_ and CHF_3_ plasma deposition^21,22^ and direct fluorination at elevated temperature.^23^ While both methods produce chemically bonded, uniform layers, they rely on energy-intensive and hazardous processes. Additionally, Gallo Stampino et al. explored the use of perfluoropolyether derivates through dip coating and thermal treatments for radical formation and grafting.^24^ Thomas et al.^25^ investigated electrochemical grafting, an energy-efficient one-step process.^26^ Hydrophobization of GDL was achieved by grafting organic molecules bearing hydrophobic −CF_3_ functionalities via diazonium salt reduction. Although these approaches enhanced coating uniformity and durability, they still depend on fluorinated compounds. Recognizing the urgent need for fluorine-free alternatives, several research groups have begun investigating other promising materials.

**Figure 1:**
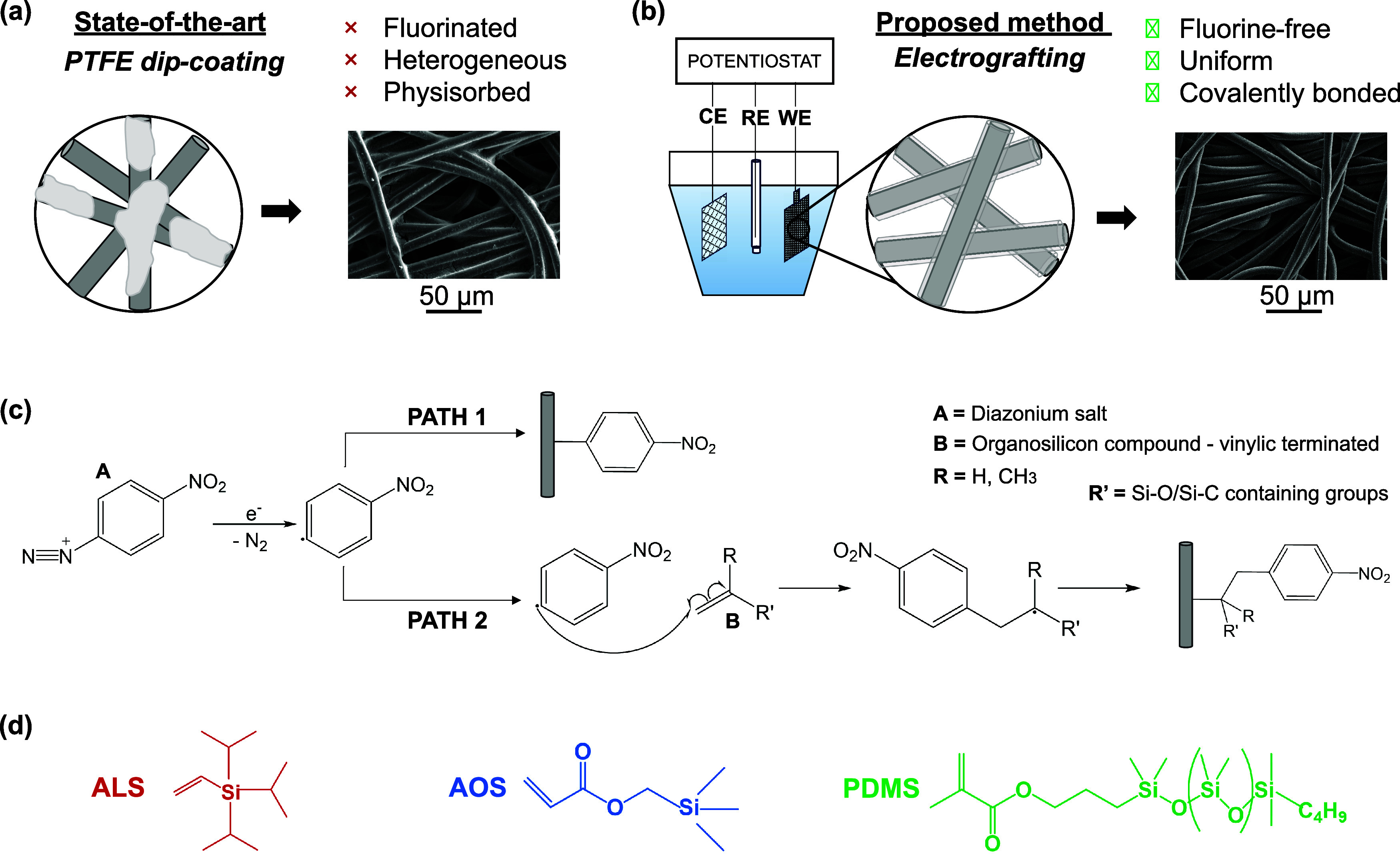
Conceptual overview of the study, illustrating our strategy to replace conventional PTFE-based dip-coating methods with energy-efficient electrografting of organosilicons: (a) schematic and SEM image highlighting the limitations of the state-of-the-art material; (b) schematic and SEM image of the newly developed material with improved properties; (c) proposed radical chain reaction mechanism initiated by the electrochemical reduction of a diazonium salt; (d) chemical structures of the vinyl-terminated organosilicon compounds investigated in this work.

Sun et al. proposed replacing PTFE with polydimethylsiloxane (PDMS) for the hydrophobization of the GDL while retaining the conventional dip-coating method.^27^ The resulting materials exhibited superhydrophobic behavior, leading to improved liquid water management, enhanced gas transport, and overall better fuel cell performance. However, the coating distribution remained uneven, highlighting the inherent limitations of the dip-coating approach in achieving a uniform surface modification. Wang et al.^28^ investigated the manufacturing of composite materials incorporating silica particles and PDMS. Silica particles were employed to modulate the surface roughness and pore size of the GDL, while PDMS contributed its intrinsically low surface energy. The resulting composite exhibited an external water contact angle of 162 ± 2° and a sliding angle of 5° while maintaining hydrophilic internal pores. This dual wettability facilitated efficient water distribution within the GDL, thereby enhancing the overall cell performance. However, the coating lacked long-term stability as its adhesion to the substrate relied solely on physical adsorption. Ko et al.^29^ introduced a hydrophobic SiOx-C:H nanolayer deposited via radio-frequency plasma-enhanced chemical vapor deposition using hexamethyldisiloxane as the precursor. This nanolayer preserved the carbon fiber morphology and porosity while increasing the external contact angle, reducing contact angle hysteresis, and suppressing water condensation. These improvements resulted in significantly enhanced fuel cell performance, but the method is energy-intensive, which challenges its economic scalability. Building on previous efforts, we introduce a novel strategy designed to address the key unresolved challenges in hydrophobizing porous media for electrochemical technologies. Our approach is conceived to be simple, scalable, and cost-effective; to form durable covalent bonds uniformly throughout complex 3D architectures without compromising pore accessibility; and to offer broad molecular versatility for functional customization (Figure 1b).

Here, we explore electrochemical grafting, a versatile surface modification technique that leverages electrochemical reduction to covalently attach hydrophobic organosilicon molecules to the conductive surfaces. This approach capitalizes on the reduction of 4-nitrobenzodiazonium tetrafluoroborate to generate aryl radicals, which start a chain reaction. The generated radicals can either directly attack the carbon surface (as depicted in Figure 1c, PATH 1) or react with the vinylic termination of the organosilicon compounds and generate new radical species that can also attack the substrate (as depicted in Figure 1c, PATH 2). Upon termination of the reaction, the surface bears functionalities derived from both the diazonium compound and the organosilicon species. Unlike the traditional electrografting of vinylics, this reaction mechanism does not require a controlled inert atmosphere, simplifying the process. Moreover, it operates at less negative reducing potential (vinylics typically require −2.1 to −2.6 V/SCE), enabling the grafting of functionalities that are otherwise inaccessible, such as groups with higher reduction potential than vinyl groups (e.g., hydroxyl groups).^26^ Therefore, it opens up the possibility to easily graft a broad spectrum of molecules, including hydrophobic species that do not contain fluorine (such as organosilicon compounds), providing a promising pathway for developing a new generation of fluorine-free, hydrophobic coatings. We envision that the resulting covalent bonds enhance the stability and durability of the coating while preserving the porous structure of the GDL, thereby optimizing mass transport and mitigating flooding. We investigated the electrografting of three distinct organosilicon-based molecules allyltriisopropylsilane (ALS), acryloxymethyl­trimethylsilane (AOS), and monomethacryl­oxypropyl-terminated polydimethylsiloxane (PDMS) to systematically tailor surface energy and hydrophobicity. The molecular structures are shown in Figure 1d. These three molecules were chosen to explore how the reactivity of the vinylic group in the electrografting process is influenced by different substituents. Specifically, we selected an allyl, an acryloxy, and a methacryloxy group to examine the effects of increasing substitution around the reactive double bond. Moreover, we selected silanes and siloxanes with varying degrees of steric hindrance to asses their role during the reaction and ultimately to evaluate the influence of these groups on the resulting wetting properties.

In this work, we first study the electrografting mechanism by performing cyclic voltammetry in a nonaqueous electrolyte with the diazonium salt and one of the three organosilicons. Second, we assess the coating uniformity and intrinsic wettability by using two model redox probes. Third, we characterize the chemical surface state with X-ray photoelectron spectroscopy. Fourth, we assess the wettability and determine the solid surface energy of the modified surfaces, including a detailed breakdown into polar and dispersive components. Finally, we tested the novel materials in polymer electrolyte fuel cells and correlate their chemical composition to cell performance. Through systematic surface and electrochemical characterization, we demonstrate that the electrografted coatings significantly reduce the solid surface energy of the carbon surfaces, achieving water contact angles comparable to those of PTFE-treated materials and competitive performance in fuel cells.

## Materials and Methods

### Chemicals and Materials

Acetonitrile (CH_3_CN, Merck, ≥99.9 %), tetraethylammonium tetrafluoroborate (TEABF_4_, Sigma-Aldrich, 99 %), silver nitrate (AgNO_3_, ≥99 %, ACS reagent), allyltriisopropylsilane (ALS, C_11_H_24_Si, CAS 24400-84-8, Gelest, 95 %), acryloxymethyltrymethylsilane (AOS, C_7_H_13_O_2_Si, CAS 67186-35-0, Gelest, 95-100 %), monomethacryl­oxypropyl terminated polydimethylsiloxane (PDMS, CAS 146632-07-7, Gelest, 95-100 %), 4-nitrobenzenediazonium tetrafluoroborate (C_6_H_4_N_3_O_2_·BF_4_, Sigma-Aldrich, 97 %), potassium chloride (KCl, Sigma-Aldrich, 99 %), potassium ferricyanide (K_3_[Fe­(CN)_6_], Sigma-Aldrich, 99 %), 4-methylcathecol (CH_3_C_6_H_3_-1,2-(OH)_2_, Sigma-Aldrich, ≥95 %) were used without further purification. Ultrapure water was used for the electrochemical experiments (18.2 MΩ, Millipore water purification system direct-Q3). A 1 cm × 1 cm glassy vitreous carbon electrode piece (GC, >99.9 %, Redoxme), 1 mm thick, was used for carrying out the modifications and studying their surface energy. Prior to modifications, the GC was polished to a mirror finish with 0.05 μm alumina slurry on a polishing pad. A commercial GDL without hydrophobic treatment (Freudenberg H15, QuinTech), with a nominal thickness of 140 μm, was used for the modifications and the rest of the ex situ and in situ characterization as received.

### Electrografting

The commercial gas diffusion layer Freudenberg H15 (FH15) was cut into 2.5 cm × 2.5 cm pieces (∼6.25 cm^2^) for fuel cell testing and into 2 cm × 2 cm pieces (∼4 cm^2^) for ex-situ characterization. Electrochemical grafting was performed by using a standard three-electrode setup. The GDL was used as working electrode, a silver/silver nitrate electrode (a silver wire was immersed in a self-made 0.1 M TEABF_4_ and 0.01 M AgNO_3_ in acetonitrile solution) was used as reference electrode, and a platinum mesh (Pt, 99 %, ≈4 cm^2^) was used as counter electrode. The Pt-mesh was flame-annealed before each experiment to eliminate possible contaminations. An acetonitrile solution containing 0.1 M supporting electrolyte (TEABF_4_), 1 mM diazonium salt (4-nitrobenzenediazonium tetrafluoroborate, 18 mg), and 10 mM one of the three organosilicon compounds, respectively, was prepared and used as an electrografting solution. Acetonitrile was selected as the solvent in order to assure complete wetting of the substrate and maximise the electrochemically active surface area for the modification. A glass electrochemistry cell (Metrohm AG, 100 mL volume) was filled with 80 mL of the previously described solution and the electrografting experiments were performed with a BioLogic VMP-300 potentiostat. The procedure consisted of 15 cycles of cyclic voltammetry, where the potential was swept from 0.5 to −1.0 V (vs Ag/AgNO_3_) with a scan rate of 100 mV/s and with automatic iR compensation (solution resistance extracted at 100 kHz and 20 mV sinus amplitude, 85 % compensation applied). It is important to note that under the applied conditions, the tetrafluoroborate anion coming from the commercial diazonium salt is typically stable.^30^ Upon reduction and formation of the aryl radical, the tetrafluoroborate remains in solution as a free anion, functioning as the counterion to preserve charge neutrality. After modification, the substrates were extensively rinsed with ultrapure water and 2-propanol in order to wash off the unreacted chemicals.

### Electrochemical Test with Redox Probes

Cyclic voltammetry using reversible redox probes was performed to correlate the charge transfer current to the electrochemically accessible surface area of the electrodes in an aqueous electrolyte. We anticipate this technique to provide semiquantitative insights into the degree of grafting and the conformality of the coatings, serving as a proxy of the wettability of the substrates. Notably, hydrophobic probe molecules tend to exhibit stronger interactions with hydrophobic coatings, whereas hydrophilic probes interact more weakly, resulting in lower or negligible charge transfer when compared to their counterparts.^31,32^ Accordingly, we selected and compared a hydrophilic probe, K_3_[Fe­(CN)_6_], and a hydrophobic probe, 4-methylcathecol (4-MC). A standard three-electrode setup was used for this experiment. The unmodified or modified GDL was used as the working electrode, a silver/silver chloride electrode (BASi, Ag/AgCl in 3 M KCl) was used as the reference, and a Pt-mesh was used as the counter. Two aqueous KCl 1.0 M solutions containing either 1 mM K_3_[Fe­(CN)_6_] or 1 mM 4-methylcathecol, respectively, were used as electrolytes. In this case, an aqueous-based electrolyte was chosen to facilitate the assessment of the hydrophobic–hydrophilic interactions between the probes and the coatings. A glass electrochemistry cell was filled with 80 mL of one or the other electrolyte, and the experiment was carried out with a BioLogic VMP-300 potentiostat. In this case, the five cycles of cyclic voltammetry were performed at a scan rate of 50 mV/s from −0.2 to 0.6 V (vs Ag/AgCl) for the solution containing [Fe­(CN)_6_]^3+^ and from −0.2 to 1.0 V (vs Ag/AgCl) for the solution containing 4-MC.

### X-ray Photoelectron Spectroscopy

The surface functionalities were determined using a Thermo Scientific K-alpha X-ray photoelectron spectrometer equipped with a monochromatic small-spot X-ray source and a 180° double focusing hemispherical analyzer with a 128-channel detector. An aluminum anode (Al Kα = 1486.6 eV) source operating at 72 W and a spot size of 400 μm were used to obtain the spectra. Survey scans were measured at a constant pass energy of 200 eV and region scans were measured at 50 eV. The background pressure was set at 2 × 10^–8^ mbar, and during the measurement, it rose to 4 × 10^–7^ mbar as a result of injecting argon ions into the chamber. For etching experiments, samples were exposed to 1 keV Ar^+^ ions with a >1 μA beam current, directed from an EX06 ion source situated within the XPS chamber. The etch area was approximately 5 times the X-ray spot-size. Samples were exposed to the ion beam for 1 min, after which a snapshot of the Si 2p peak was recorded. This sequence was repeated 60 times.

### Contact Angle Measurements

External contact angle measurements were performed on an unmodified GDL, a dip-coated GDL with a 10 wt % PTFE loading, and electrografted GDLs. The contact angle measurements were performed at room temperature with a drop shape analyzer for standard automatic contact angle measurements (KRÜSS DSA30S). The instrument was equipped with a high resolution (1920 × 1200 px) and high speed (2300 fps) camera from Basler. The software ADVANCE AD3221 was utilized for the analysis. The droplets were ejected at a fixed distance from the surface and gradually brought closer. Upon first visible contact between the droplet and the surface, the needle was withdrawn, and after stabilization, the measurements were done. The reported contact angles were estimated as the average value of three different measurements of 2 μL of water droplets on porous substrates and of 5 μL of liquid droplets on the flat substrates.^33^

### Surface Energy Measurements

Surface energy is a fundamental parameter for evaluating molecular interactions between liquids and solid surfaces, as it allows a prediction of how a solid will interact with any liquid of known physicochemical properties. A high solid surface energy indicates that the interfaces between solids and air are thermodynamically unfavorable, meaning that high surface energy solids are readily wetted by liquids, as the liquid will replace the solid–air interface with a more favorable solid–liquid interface.^34^ Conversely, low surface energy solids are poorly wetted by most liquids. To obtain surface energy values, contact angle measurements were performed on unmodified GC, electrografted GC, and flat PTFE sheets for comparison. Glassy carbon was used in these measurements as a model substrate due to its quasi-flat surface which more adequately satisfy the conditions of perfectly smooth surfaces required by Young’s equation. The same electrografting procedure as described above was carried out for the modification of the glassy carbon pieces (1 cm × 1 cm). All the materials were tested at room temperature with five different solvents: methanol, ethanol, *n*-propanol, acetone, and water. The contact angle was again estimated as the average value of three different measurements of 5 μL of liquid droplets on each sample. By using the Owens–Wendt theory,^35^ which accounts both for van der Waals and other non-site specific interactions (dispersive), and for dipole–dipole, dipole induced–dipole, hydrogen bonding, and other site-specific interactions (polar) between solid surfaces and liquids, the solid surface energy can be calculated along with its individual components. The method relies on known surface tension values of the liquids used and the measured contact angle values of the solid surfaces. These values are reported in the Supporting Information in Table S1 and Table S2, respectively.

Thus, the solid–vapor γ_SV_ and liquid–vapor γ_LV_ interphases are expressed by the following equations:

$${\gamma_{\mathrm{SV}}=\gamma }_{\mathrm{SV}}^{d}+\gamma_{\mathrm{SV}}^{p}$$

$${\gamma_{\mathrm{LV}}=\gamma }_{\mathrm{LV}}^{d}+\gamma_{\mathrm{LV}}^{p}$$

Combining Good–Fowkes’ eq 1 with Young’s eq 2,

1 $$\gamma_{\mathrm{SL}}=\gamma_{\mathrm{SV}}+\gamma_{\mathrm{LV}}-2\left(\sqrt{\gamma_{\mathrm{SV}}^{d}\gamma_{\mathrm{LV}}^{d}}+\sqrt{\gamma_{\mathrm{SV}}^{p}\gamma_{\mathrm{LV}}^{p}}\right)$$

2 $$\gamma_{LV}\cos\theta =\gamma_{SV}-\gamma_{SL}$$

yields the linear relation [Disp-formula eq3],

3 $$\left(1+\cos\theta \right)\frac{\gamma_{\mathrm{LV}}}{2\sqrt{\gamma_{\mathrm{LV}}^{d}}}=\sqrt{\gamma_{\mathrm{SV}}^{d}}+\sqrt{\frac{\gamma_{\mathrm{LV}}^{p}}{\gamma_{\mathrm{LV}}^{d}}}\sqrt{\gamma_{\mathrm{SV}}^{p}}$$

where θ is the contact angle between liquid and solid. The two unknown components of solid surface energy *γ*_SV_^d^ and *γ*_SV_^p^ can then be determined by substituting the variables with the known values in eq 3.

A plot of $\left(1+\cos\theta \right)\left(\left(\gamma_{\mathrm{LV}}^{p}+\gamma_{\mathrm{LV}}^{d}\right)/2\sqrt{\gamma_{\mathrm{LV}}^{d}}\right)$ versus $\sqrt{\gamma_{\mathrm{LV}}^{p}/\gamma_{\mathrm{LV}}^{d}}$ for the different liquids yields the dispersive component γ_SV_^d^ (square of the *y*-intercept), the polar component γ_SV_^p^ (square of the slope), and consequently the surface tension of the solid–vapor interface γ_SV_, or in other terms, the solid surface energy.^36^ The corresponding plots used to extract these parameters are provided in Figure S7.

### Membrane Electrode Assembly and Single Fuel Cell Tests

Fuel cell tests were performed in 5 cm^2^ active area single cell hardware (qCf FC25/100 V 2.0 LC, Baltic Fuel Cells GmbH) with graphite flow fields comprising mirror-symmetrical flow patterns for the anode and cathode. The flow fields consisted of 22 parallel channels with a channel and land width of 0.3 mm and 0.6 mm, respectively, and a channel depth of 0.5 mm. In all experiments, a homemade PTFE dip-coated GDL (10 wt %) was used at the anode diffusion medium. The cathode diffusion medium used was either a PTFE dip-coated GDL or one of the three electrografted GDLs. To focus on understanding the role of chemical modifications on the GDL and to avoid additional influences from other components, we elected not to use MPL in the present study. The catalyst coated membranes (CCMs) were received from EKPO Fuel Cell Technology (EKPO) and consisted of a Nafion membrane (GORE-SELECT M788.12), an anode catalyst loading of 0.1 mgPt/cm^2^, and a cathode catalyst loading of 0.4 mgPt/cm^2^. The square active electrode area was 5.0 cm^2^, while the GDL cuttings were 5.8 cm^2^ (e.g., when perfectly aligned, the GDL extended 0.9 mm beyond the active area) in order to ensure that the GDL was always covering the active area within the errors of alignment. A GDL compression of ∼20 % with respect to its initial thickness was targeted in the cell fixture for a sufficient contact pressure between the layers. To realize this, polyethylene naphthalene foils with a polyester resin based heat seal adhesive (CMC 61325, CMC Klebetechnik GmbH), in the thickness of 0.025 mm, were placed on the anode and cathode side as gaskets and achieve the desired compression. Last, the CCM was sandwiched between the two GDLs and the flow fields, and the cell was connected to a FuelCon Evaluator-C 70240 test station equipped with a Biologic HCP-803 potentiostat (voltage range of ±5 V at 1 A and ±3 V at 80 A, current range of ±80 A, EIS capability from 10 kHz to 10 μHz). Prior to fuel cell testing, we applied a break-in procedure by stepping the voltage under hydrogen (constant flow of 500 nccm) and air (constant flow of 5000 nccm) at either *T*_cell_ = 50 °C or *T*_cell_ = 80 °C, *p*_abs_ = 150 kPa, and full humidification in the following sequence until stable performance was reached: 0.6 V for 1 min, 0.3 V for 1 min, and open circuit voltage (OCV) for 1 min, repeated 20 times. Potentiostatically controlled polarization curves were recorded by stepping the voltage from the OCV to 0.3 V, in steps of 0.05 V, under constant flow rates of 500 nccm of hydrogen (5.0 quality) and 5000 nccm of air, constant *p*_abs_ of 150 kPa, constant relative humidity of 100 %, and two different temperature conditions, *T*_cell_ = 50 °C and *T*_cell_ = 80 °C. Each voltage was maintained for 5 min, with data points recorded every second. To plot the polarization curves, the average of the data collected during the final minute (after 4 min of stabilization) was used. The error bars represent the standard deviation calculated from measurements obtained from two indipendent samples. After each potential step, an impedance spectrum was recorded from 100 kHz to 10 Hz with a perturbation voltage of 20 mV, with 8 points per decade in logarithmic spacing, 3 averaged measurement per frequency, and a bandwidth of 3. The high frequency resistance (HFR) was determined from the high-frequency intercept on the real axis in the Nyquist representation (imaginary part vs real part of impedance).

## Results and Discussion

### Electrografting Footprint

We set out to investigate the effectiveness and underlying mechanism of electrografting on gas diffusion layers. The electrochemical grafting signatures of the three organosilicons are shown in Figure 2a, where all exhibit similar voltametric behavior. In the first potential cycle, spanning from 0.5 to −0.8 V vs Ag/AgNO_3_ in the reductive direction, two distinct peaks were observed: one at 0.0 V and another one at -0.5 V. These peaks correspond to the reduction of the diazonium salt into a free radical and then into a cation, respectively.^37^ As the number of voltage cycles increases, the current intensity decreases and the reduction peaks shift to lower potentials. This behavior is characteristic of diazonium salts, where the formation of an organic passivating layer on the electrode surface progressively alters the reduction kinetics, eventually blocking the access of diazonium species to the electrode surface.^26,38^ Consequently, the disappearance of the peaks indicates surface saturation. Furthermore, the fact that both the main and secondary peaks occur at the same voltages for all three chemistries suggested that the reaction mechanism is consistent across them.

**Figure 2:**
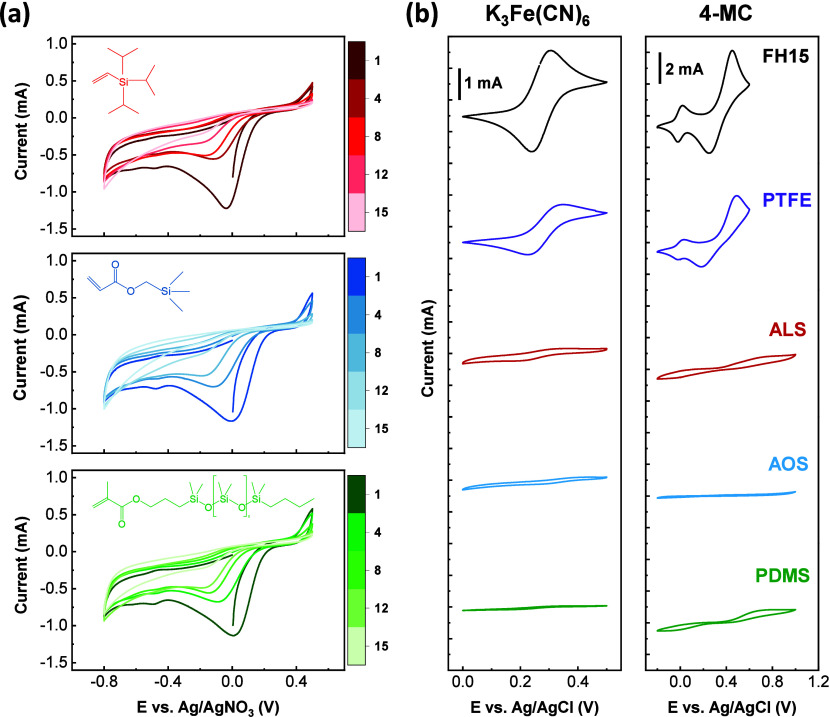
Electrochemical grafting footprint and electrochemical characterization with redox probes of baselines and grafted material: (a) cyclic voltammetry of 4-nitrobenzenediazonium salt (1 mM) and a vinylic terminated silane/siloxane (10 mM) in acetonitrile, 15 cycles at 50 mV/s, onto carbon fibers paper (Freudenberg H15); (b) cyclic voltammetry of ferricyanide (1 mM) on the left and 4-methylcathecol (1 mM) on the right in KCl 1.0 M, 5 cycles at 100 mV/s, on baselines and grafted materials.

We hypothesize that, first, the diazonium salt is electrochemically reduced into a free radical (at 0.0 V vs Ag/AgNO_3_). Then, this free radical can either form a covalent bond with the carbonaceous substrate or react with the vinylic termination of the organic compound in solution (Figure 1d). As a result, a radical species carrying the hydrophobic functionality is generated, which can also form covalent bonds with the carbonaceous electrode. Since the reduction of the vinylic functionality occurs via a chemical process rather than an electrochemical one, it does not produce a signal in the voltammogram. The similarity in current intensity is likely due to the comparable available surface area of the electrodes, as the same material and geometric electrode area were used for all modifications. This hypothesis is further supported by the electrografting footprint of the diazonium salt alone. As shown in Figure S1, the cyclic voltammogram for the grafting of nitrobenzene diazonium salt alone (NBD) displays essentially the same features as those observed for the organosilicon-containing electrolytes. Furthermore, employing a nonaqueous electrolyte (here acetonitrile) significantly enhances access to the inner porous structure compared to the aqueous electrolyte. This was evidenced by double layer capacitance measurements, which showed values of 660 μF in 0.1 M TEABF_4_ in acetonitrile versus 150 μF in 0.5 M HCl (Figure S2).

### Redox Probes To Interrogate Coating Uniformity and Wettability

To further validate the effectiveness of the electrografting procedure and assess the conformality of the grafted layers, we performed cyclic voltammetry in an aqueous electrolyte solution containing a redox-active compound. We employ hydrophilic (K_3_[Fe­(CN)_6_]) and hydrophobic (4-methylcathecol) redox probes to interrogate the electrochemical state of the surface and the uniformity of the coating. The selection of probes with differing water affinities accounts for the fact that hydrophobic/hydrophilic interactions between the probe and the coating layer influence the measurements. Therefore, we anticipate that this approach can serve as a proxy of the relative hydrophobicity of the grafted layers.

As shown in Figure 2b, the experiment was conducted on the FH15 electrode prior to any treatment, after dip-coating with PTFE (10 wt % loading), and after electrografting. On the left side of the figure, the results for (K_3_[Fe­(CN)_6_]) are presented. The untreated electrode exhibits the characteristic “duck shape” voltammogram, indicative of a reversible redox reaction, with distinct oxidation and reduction peaks at 0.30 and 0.24 V, respectively.^39^ After PTFE dip-coating, the material shows distinct peaks but are slightly shifted (oxidation peak at 0.34 V and reduction peak 0.22 V) and with reduced current intensity. This result suggests that the PTFE coating reduces the available electrode surface for electron transfer and alters the reaction kinetics, although it does not completely block the charge transfer process. This effect can likely be attributed to the heterogeneity of the coating that leaves part of the electrode surface exposed and not covered by the insulating PTFE layer, as evidenced by the SEM and EDX images in Figure S3.

In contrast, the electrografted electrodes show very weak or no redox peaks, indicating complete coverage of the electrode surface. The electrochemical behavior of the electrografted electrodes can be better seen in the zoomed-in voltammograms in Figure S4. For comparison, Figure S4 also shows the behavior of the substrate grafted with NBD alone, highlighting that the presence of organosilicons in the coated layer has a significant influence on the electrochemical properties. We hypothesize that the covalently bonded organic layer formed around the fibers acts as a barrier to the redox reaction of the hydrophilic ferricyanide. On the right side of Figure 2b, the cyclic voltammetry results for 4-methylcatechol are presented. The trends observed closely resemble those seen with ferricyanide. The untreated electrode displays two oxidation peaks at 0.01 and 0.45 V and two reduction peaks at 0.25 and 0.02 V, consistent with literature values.^40^ In contrast, the PTFE-coated electrode exhibits slightly shifted peaks (oxidation at 0.03 V and 0.49 V and reduction at 0.18 V and 0.02 V) along with reduced current intensity. Interestingly, for the electrografted electrodes, one of the two redox peaks is completely suppressed, while the other shows a reduced intensity and a notable potential shift. This behavior suggests that the interaction between a hydrophobic redox probe and a hydrophobic organic layer may facilitate electron transfer more effectively than the interaction between a hydrophilic probe and a hydrophobic layer under similar conditions. In fact, redox probes undergo electron transfer at a distance from the electrode that is defined by the average thickness of the organic layer formed on the substrate.^31^ It can occur through a long-range process across the organic layer and/or via structural defect sites, such as pinholes.^41^ Importantly, the barrier properties of the organic film are highly dependent on the solvation properties and hydrophobicity of the redox probe, highlighting the complex interplay between the surface chemistry and interfacial electron transfer dynamics.

Hydrophobic and hydrophilic interactions between the redox probe and the organic layer significantly influence the electron transfer rate. In aqueous media, hydrophilic probes are typically surrounded by a hydration shell that maintains a greater distance from the electrode surface, whereas hydrophobic probes can approach more closely, facilitating faster electron transfer. Additionally, hydrogen bonds can be formed at the probe/layer interface, further increasing the stability of the probe and hindering electron transfer. Consequently, hydrophilic probes tend to have limited access to hydrophobic layers, while hydrophobic probes are less hindered. Conversely, the opposite is observed when hydrophilic layers are used.^42,31^ In this specific case, the hydrophobic probe (4-MC) also exhibits some degree of hydration due to its two hydroxyl groups, which can form hydrogen bonds with surrounding water molecules. However, this hydrogen-bonded solvation shell is relatively flexible and more prone to rearrangement.^43^ In contrast, the solvation shell around the hydrophilic probe (K_3_[Fe­(CN)_6_]) is dominated by stronger, more rigid electrostatic and polar interactions, making it less dynamic and more stable.^44^ Therefore, initial evidence of the hydrophobic nature of the electrografted coatings was provided by this test. To further validate these findings, we replicated the experiments on model flat substrates (i.e., glassy carbon). As shown in Figure S5, the electrografted surfaces significantly hinder electron transfer, with a more pronounced barrier effect observed for the hydrophilic redox probe compared with the hydrophobic one. In contrast, the PTFE-coated substrate exhibits only minor deviations from the untreated surface, consistent with the trends observed in the porous gas diffusion layers.

### Surface Chemical Composition

We then characterized the surface functionalities by means of XPS. The survey spectra of the untreated and electrografted carbon electrodes are shown in Figure 3a, and the corresponding elemental distributions are shown in Figure 3b. The spectra reveal distinct silicon signals for the electrografted material, which are absent in the pristine electrode, thus confirming the presence of a coating. Additionally, the pristine electrode shows a low amount of oxygen and nitrogen (with >97 atom % carbon), whereas the electrografted materials contain approximately 9% nitrogen and 19% oxygen. These findings align with expectations for grafting a molecule containing a nitro group (−NO_2_), where the number of oxygen atoms is twice that of nitrogen. Further evidence of the presence of the nitro group in the electrografted samples is provided by the N 1s high-resolution spectra shown in Figure 3c. The main peak at 405 eV corresponds to the −NO_2_ group, while smaller peaks at 402.4, 400, and 398 eV are attributed to the π–π* shake-up, pyrrolic nitrogen and −NH_3_ group, and pyridine nitrogen, respectively.^45^ On the right side of Figure 3c, the high-resolution Si 2p spectra are shown. Firstly, we observe that the signal intensity increases for the three samples, going from ALS to AOS to PDMS, suggesting that the PDMS-grafted material contains the highest amount of Si atom. Secondly, upon deconvoluting the Si 2p peaks, we see the characteristic presence of the Si 2p_1/2_ and Si 2p_3/2_ peaks in the typical 1:2 intensity ratio. Core levels in XPS use the nomenclature *nlj* where *n* is the principal quantum number, *l* is the angular momentum, and *j* = l + *s* (where *s* is the spin angular momentum and can be ±1/2). All orbital levels, except *s* (*l* = 0), exhibit spin-orbit splitting, producing a doublet with the two possible states having different binding energy. The relative intensities of these peaks correspond to the degeneracy of each spin state: for instance, in the 2p spectrum, the area ratio of the 2p_1/2_ to 2p_3/2_ peaks is 1:2, corresponding to two electrons in the 2p_1/2_ state and four in the 2p_3/2_ state.^46^ These peaks shift slightly depending on the bonding environment of the Si atoms.^47–49^

**Figure 3:**
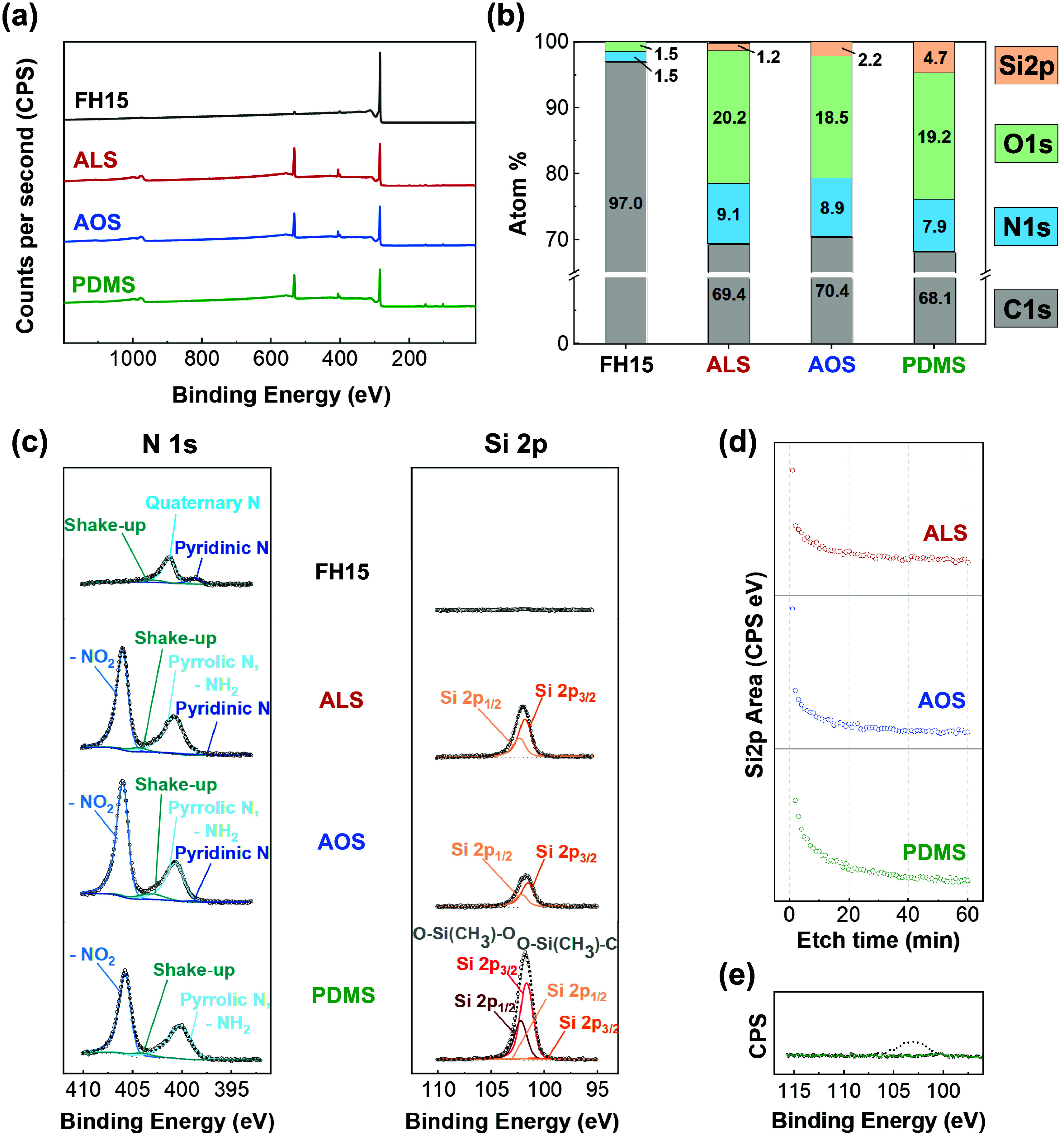
XPS of the electrode surfaces to quantify the functional groups: (a) XPS survey spectra of the untreated FH15 and of the three electrografted electrodes; (b) elemental compositions extracted from the survey spectra; (c) N 1s and Si 2p high resolution spectra before etching and Si 2p after etching (etching is performed at 1 keV with a monochromatic Ar^+^ ion gun within the XPS chamber); (d) Si 2p peak area as a function of etching time; (e) Si 2p peak of the PDMS electrografted GC, before etching and after 1 cycle of etching.

To estimate the thickness of the grafted layer, XPS depth profiling was performed. The Si 2p signal was measured after exposing the sample to an Ar^+^ beam with 1 keV energy, etched at 1 min intervals for 60 cycles. The total area under the Si 2p peak was then plotted against the etching time, as shown in Figure 3d, and the Si 2p high resolution spectra before and after etching are reported in Figure S6. The Si 2p signal drops to baseline levels after 15–20 min, indicating that the carbon fiber surface is reached. Although each coating responds differently to the Ar^+^ beam, leading to variations in etching times, we observed that a similar number of etching cycles are required for the complete removal of the three different coatings. However, these results were likely significantly influenced by the porous nature of the substrate and the hindered penetration of the X-rays and Ar ion beam. To further assess coating thickness, the same experiment was repeated on planar electrografted glassy carbon electrodes (Figure 3e). We observed that on a flat substrate, only one etching cycle is sufficient to remove the coating, suggesting that the thickness of the organic layers we formed is likely below 10 nm. In fact, according to literature, PDMS etches at 10–100 nm/min during Ar-ion etching, depending on factors like ion energy, pressure, and substrate temperature.^35^ Nevertheless, it is important to note that layer thicknesses can significantly influence surface properties. Therefore, when interpreting the wettability and electrochemical data, one should consider that the observed effects may not arise solely from surface chemistry.

### External Wettability and Surface Energy

To assess the wettability of the electrografted materials, we performed external water contact angle measurements using the sessile-drop method on both 2D and 3D substrates (Figure 4a, numerical values in Table S3 and Table S4). The electrografted materials were compared to the pristine GC and a PTFE sheet for the 2D substrates and to the pristine GDL and a 10 wt % PTFE dip-coated one for the 3D substrates. The pristine GC and the PTFE sheet exhibit contact angle values of 64 ± 1° and 110 ± 1°, respectively, while the electrografted GC materials show values of 86 ± 1° for ALS, 85 ± 7° for AOS, and 92 ± 5° for PDMS. Although the organosilicon-based coatings did not achieve the high water repellency of PTFE, they demonstrated significantly higher repellency than the untreated carbon. Furthermore, we show that through molecular engineering, the resulting contact angles can be tuned, which is appealing for a range of electrochemical applications. A similar trend was observed for the porous GDL material, where the pristine electrode and the PTFE dip-coated electrode feature contact angles of 130 ± 4° and 150 ± 2°, respectively, while the electrografted electrodes show values of 132 ± 3° for ALS, 132 ± 6° for AOS, and 140 ± 5° for PDMS. Thus, we observed that all the porous substrates are hydrophobic, with the PDMS showing the highest hydrophobicity among the electrografted ones and closely approaching that of the PTFE dip-coated one. This can be attributed to the fact that although the Si–O bond within the polymer backbone is polar, the overall surface is rendered relatively apolar due to the repeating Si–O units and the surface exposure of apolar substituents such as the methyl groups (−CH_3_). This combination results in a surface that preferentially repels water, enhancing the hydrophobic character of PDMS compared to the other coatings.^50^

**Figure 4:**
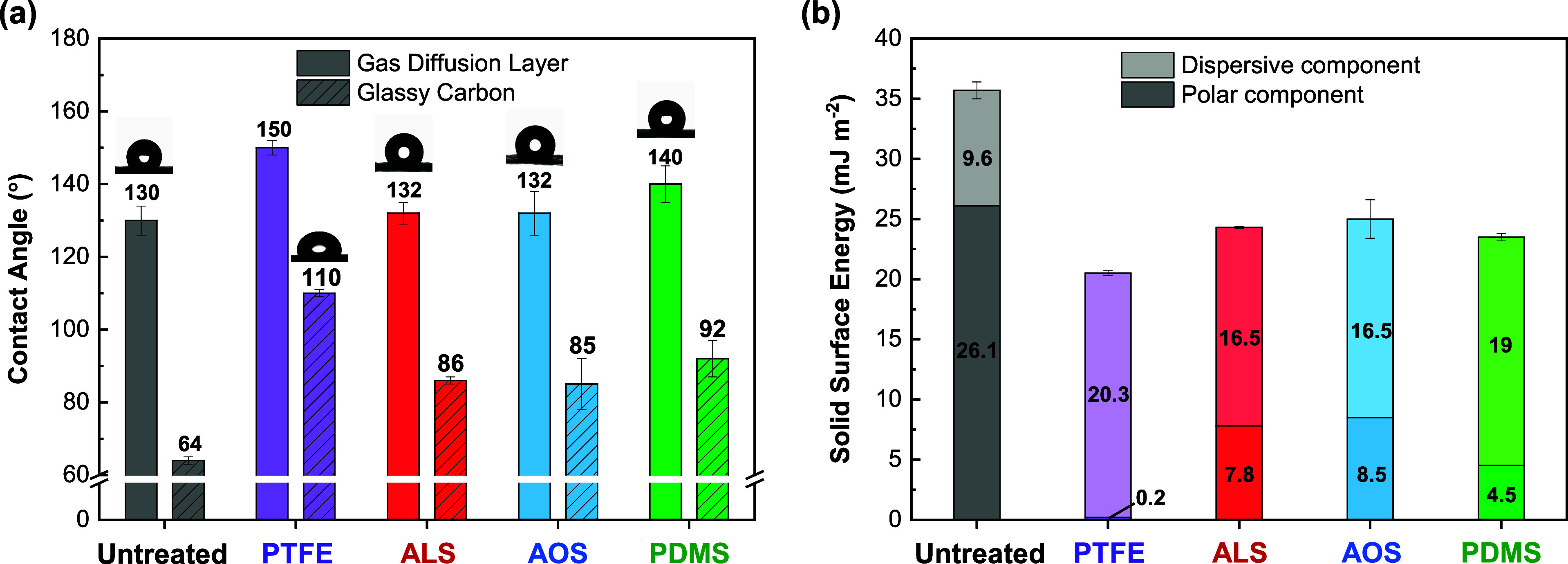
Water contact angles and solid surface energy values to assess wetting properties. (a) Contact angles of 2 μL water droplets onto untreated, PTFE dip-coated and electrografted GDL (FH15) in full colours; contact angles of 5 μL water droplets onto untreated GC, PTFE flat sheet, and electrografted GC in stripe pattern. (b) Solid surface energy values, with polar and dispersive components, were measured on 2D substrates. Untreated and electrografted GC (1 cm × 1 cm) and on PTFE flat sheet.

While external contact angles offer insights into surface wetting behavior, they are strongly influenced by surface roughness and may not reliably predict water management performance within porous structures.^51^ To gain more meaningful insights, significant efforts were made to measure advancing and receding contact angle and thereby determine the materials hysteresis.^52^ However, we were unable to obtain reliable data, since even a small number of surface heterogeneities can induce droplet pinning and strongly affect the measurements, particularly on nonsmooth surfaces that deviate from the assumptions of Young’s model.^53^ Similar challenges have been reported for fuel cell gas diffusion layers.^54–56^ Therefore, to better understand the intrinsic wetting properties of the modified surfaces, we quantified the solid surface energy on smooth surfaces. Given the difficulty of performing accurate surface energy measurements on porous substrates, we conducted these experiments on two-dimensional GC electrodes. Although GC differs from carbon fiber papers, it offers a useful comparative baseline to assess the relative changes induced by surface modifications. Importantly, using GC eliminates roughness effects, allowing us to isolate the contribution of surface chemistry and evaluate the intrinsic hydrophilicity or hydrophobicity of the coatings. The calculated surface energy values were consistent with the observed contact angles and are summarized in Figure 4b. Linear fits using the Owens–Wendt method are provided in Figure S7.

All three electrografted coatings exhibit reduced total surface energy relative to the pristine GC, though higher than that of PTFE.^57^ Notably, the PDMS-modified surface displays the lowest polar component among all studied samples, indicating the weakest interaction with water and thus the highest degree of water repellency. This trend aligns with our electrochemical measurements with redox probes: the PDMS coating, with the lowest polar surface energy, produced the lowest current response for the hydrophilic probe. Conversely, the AOS coating, which exhibited the lowest dispersive component, showed the lowest current response to the hydrophobic probe. Based on these results, we anticipate PDMS to offer superior hydrophobicity among the electrografted materials. Finally, we note that the characterization of the wetting properties was also performed for materials grafted with NBD alone, and the related data are included in the Supporting Information alongside the other materials. The results demonstrate that the diazonium salt alone imparts hydrophilic properties to the substrate.

### Fuel Cell Performance

We finally evaluated the performance of the novel coatings in operando in polymer electrolyte fuel cells. For clarity, a schematic of the PEFC with a magnified view of the cathode side is provided in Figure S8. Polarization curves and high-frequency resistance (HFR) values for the PTFE dip-coated and electrografted electrodes are shown in Figure 5, with all samples tested under identical gas flow, relative humidity, and pressure conditions but at different operating temperatures. The corresponding power density curves are presented in Figure S9. Figure 5a and Figure 5b display the results for the three electrografted materials at 80 °C and 50 °C, respectively. Consistent with the contact angle and surface energy measurements (where the PDMS-modified electrode showed the highest hydrophobicity), it also delivered the best fuel cell performance at both operating temperatures. In contrast, the AOS-modified material, which featured the highest polar surface energy component and thus the strongest interactions with water, shows poorer performances at 50 °C, when more liquid water is present in the cell. From these results, we concluded that the proposed hydrophobizing method enables tuning of the wetting properties of the diffusion media, thereby influencing the cell performance. Among all the electrografted materials, PDMS provides the best balance of hydrophobicity and efficiency under the tested conditions. This material was thereby compared to the state-of-the-art (10 wt % PTFE) under the same conditions (Figure 5c).

**Figure 5:**
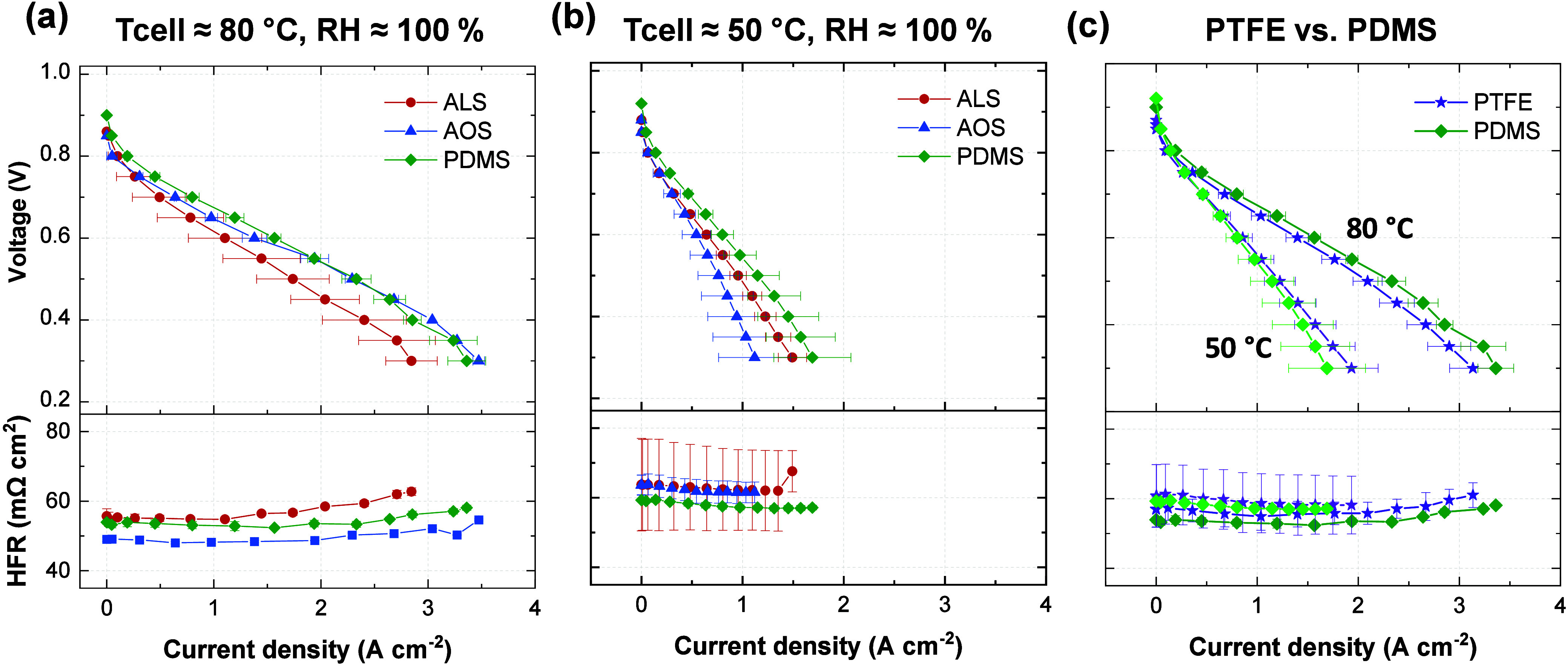
Differential-flow H_2_/air (0.5/5 nlpm) polarization curves showing cell voltage (top) and high frequency resistance (bottom) vs current density for the cathode electrografted GDLs and for the state-of-the art GDL. Operating conditions are (a) *T*_cell_ = 80 °C, RH = 100 %, *p*_abs_ = 150 kPa; (b) *T*_cell_ = 50 °C, RH = 100 %, *p*_abs_ = 150 kPa; (c) *T*_cell_ = 50 °C vs *T*_cell_ = 80 °C, RH = 100 %, *p*_abs_ = 150 kPa. The error bars represent the standard deviation of the three independent measurements.

When tested at a cell temperature of 50 °C, the PTFE dip-coated and the PDMS-modified GDLs exhibit comparable performance at low current densities. However, at higher current densities, the PTFE-coated electrode slightly outperforms its PDMS counterpart. This behavior is likely due to improved water management by the PTFE coating under high-humidity conditions. Owing to its greater hydrophobicity and lower affinity for water, PTFE more effectively mitigates electrode flooding at elevated current densities. An alternative explanation could be the heterogeneous nature of the PTFE coating, which may create preferential pathways for water removal and, thereby, enhance mass transport.

In contrast, testing at a cell temperature of 80 °C (more representative of industrial operating conditions, where less liquid water is present and the likelihood of flooding is reduced), the PDMS-electrografted GDL demonstrates superior performance. The resulting nanometer-thick layer uniformly modifies the surface without obstructing the pore volume. In comparison, PTFE dip-coating often results in agglomerates and surface clusters that partially block the porous structure, hindering gas diffusion and inducing mass transport limitations.^21^ For reference, a 30% wt.PTFE loading would reduce the porosity of a GDL (80% initial porosity) to a value of ca. 74.6% porosity. Furthermore, the conformal grafted layers do not significantly compromise the electrical conductivity of the GDL or its electronic contact with the catalyst layer or the flow field. This is evidenced by consistently lower HFR values for the PDMS-electrografted samples compared to those with dip-coated PTFE. This is a noteworthy result, as uniform coatings with nonconductive materials are typically expected to hinder electron transport; however, our findings demonstrate that this effect is negligible in the case of our electrografting strategy.

The large error bars observed in our experiments are primarily attributed to the absence of a microporous layer (MPL) on top of the GDL. The MPL typically enhances the electrical contact between the catalyst layer and the GDL, thereby reducing the ohmic resistance. Moreover, the MPL improves water manageement, minimizing the risk of flooding, which in turn results in more consistent transient current–voltage data.^58,59^ We have performed supplementary experiments to assess this hypothesis and obtained significantly smaller error bars (Figure S9) and a lower high frequency resistance (50–60 mΩ cm^2^ without MPL and 35–40 mΩ cm^2^ with MPL at 80°C and 100% RH).

In this study, we deliberately excluded MPL to isolate the effects of the surface modification on GDL, eliminating the influence of this additional layer. This choice, however, led to higher and more variable ohmic resistance and increased susceptibility to flooding, both of which contribute to the broader error margins. Furthermore, while all primary results in this work were obtained using Freudenberg substrates, repeating the functionalization on Toray carbon papers (Toray TGP-H060) yielded improved reproducibility and narrower error bars (Figure S10). These findings suggest that part of the observed variability is substrate-dependent. Nonetheless, the results clearly demonstrate the robustness of our modification strategy and its compatibility across different types of carbon-based GDLs. Most importantly, we have shown that our electrografted materials deliver performance comparable to and in some cases exceeding that of state-of-the-art PTFE-coated GDLs. This highlights a promising path forward for the development and scaling of fluorine-free alternatives. Looking ahead, we envision integrating PFAS-free microporous layers to create fully functional, environmentally benign GDLs with a competitive performance.

## Conclusion and Outlook

We have introduced a successful strategy for developing fluorine-free hydrophobic coatings for gas diffusion layers in polymer electrolyte membrane fuel cells via electrografting. By leveraging the electrochemical reduction of diazonium salts, we covalently anchored organosilicon compounds onto carbon substrates, forming conformal, durable, and water-repellent coatings. Among the three formulations tested, the PDMS-based coating exhibited surface energy characteristics comparable to PTFE (23.5 mJ/m^2^ vs 20.5 mJ/m^2^), highlighting its potential as a competitive, environmentally friendly alternative. Electrochemical testing revealed a strong correlation between the polar component of surface energy and the fuel cell performance under water-rich operating conditions. The PDMS-modified GDL consistently outperformed the other grafted molecules, particularly under high humidity, due to its enhanced hydrophobicity and superior water management. Moreover, unlike traditional PTFE dip coatings, the electrografted PDMS layer provided a conformal coating that minimized pore blockage, thereby improving gas transport and reducing mass transfer losses. Beyond the application of these novel coatings on low temperature fuel cells, the electrografting strategy presented here offers a versatile and scalable platform for functionalizing porous materials with tailored interfacial properties. Given its simplicity, conformality, and ability to introduce diverse functional groups, this method is promising for broad application in electrochemical technologies, including redox flow batteries, CO_2_ electrolyzers, and metal–air batteries. In aqueous electrochemical systems, controlled hydrophobization can reduce unwanted wetting, increase local gas availability, and improve bubble management.^60,61^ In continuous gas diffusion electrodes, wettability governs the triple phase boundary (gas–liquid–solid) and thereby influences both the activity and selectivity. While hydrophobicity helps prevent flooding, excessive hydrophobicity can produce a stable gas layer (plastron) at the solid–liquid interface, causing ionic insulation and increased resistance.^62^ Therefore, precise control over electrode wettability, as offered by our proposed method, is a critical design parameter for optimizing durability and performance of several electrochemical technologies, such as CO_2_ and N_2_ electroreduction, metal–air batteries, and related systems.^63–69^

## Supplementary Material



## Data Availability

The data presented in this study can be found in the Supporting Information or can be provided by the corresponding author upon reasonable request.

## Notes

Control experiments with NBD alone, Electorhcemical Double-Layer Capacitance measurements, Scanning Electron Microscopy images, additional cyclic voltammetries with redox probes, Si 2p region high-resolution XPS spectra before and after Ar-etching, numerical values of contantc angle measurements, Owens-Wendt linear fits, additional in operando fuel cell data (PDF)
