# Supplementary material for: Hydrophobic Diffusion Media via Electrografted Organosilicons Enable Competitive Performance in Polymer Electrolyte Fuel Cells
Source: ACS Electrochem. 2025 Nov 4;1(12):2723–35. doi: 10.1021/acselectrochem.5c00333 (PMC12683640; doi:10.1021/acselectrochem.5c00333)
Supplement: Supplementary file 1 [file ec5c00333_si_001.pdf]

Supporting Information

Hydrophobic Diffusion Media via Electrografted Organosilicons Enables Competitive Performance in Polymer Electrolyte Fuel Cells

Irene Sinisgalli, Adrian Mularczyk, Antoni Forner-Cuenca*

Electrochemical Materials and Systems, Department of Chemical Engineering and Chemistry, Eindhoven University of Technology, P.O. Box 513, 5600 MB Eindhoven, The Netherlands

*Corresponding author: a.forner.cuenca@tue.nl

Table of Contents

Electrografting footprint S4

Figure S1...... S4

Electrochemical Double Layer Capacitance...... S4

Figure S2...... S4

Scanning Electron Microscopy S5

Figure S3...... S5

Cyclic voltammetry with redox probes S6

Figure S4...... S6

Figure S5...... S6

X-Ray Photoelectron Spectroscopy S7

Figure S6...... S7

Contact angle and solid surface energy measurements S7

Table S1...... S7

Table S2 ...... S8

Table S3...... S9

Table S4...... S9

Figure S7...... S10

Table S5...... S11

Single-cell testing S12

Figure S8...... S12

Figure S9...... S132

Figure 10...... S133

Figure 11...... S134

References S15

Abbreviations

**GDL**	Gas Diffusion Layer
**GC**	Glassy Carbon
**PTFE**	Polytertafluoroethylene
**NBD**	Nitrobenzendiazonium salt
**ALS**	Allyltriisopropylsilane
**AOS**	Acryloxymethyltrimethylsilane
**PDMS**	Polydimethylsiloxane

Electrografting footprint

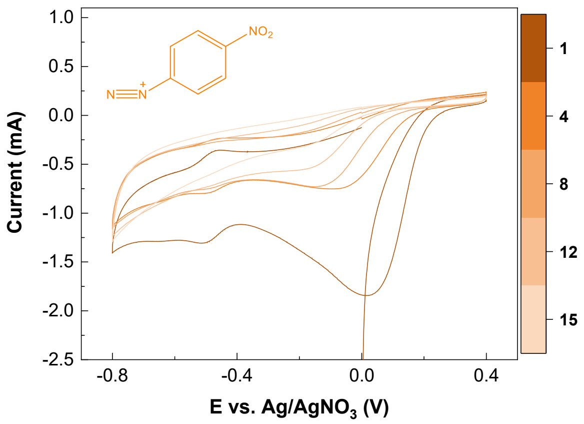

Figure S1. Cyclic voltammetry of 4-nitrobenzendiazonium salt (1 mM) in acetonitrile, 15 cycles at 50 mV/s onto the carbon fiber paper (Freudenberg H15).

Electrochemical Double Layer Capacitance

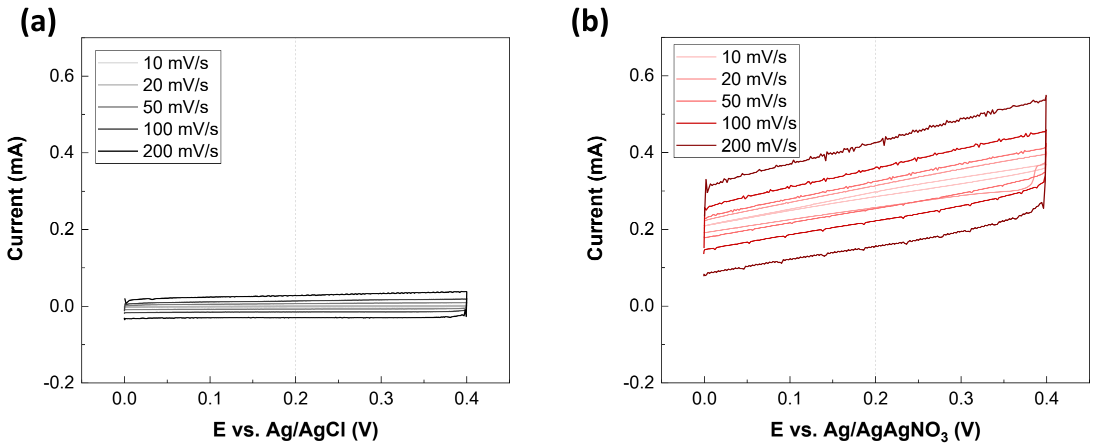

Figure S2. Cyclic voltammograms of untreated FH15 in **(a)** 0.5 M HCl and **(b)** 0.1 M tetraethylammonium tetrafluoroborate in acetonitrile in a 3-electrode cell set-up.

Scanning Electron Microscopy and Energy Dispersive X-ray Analysis

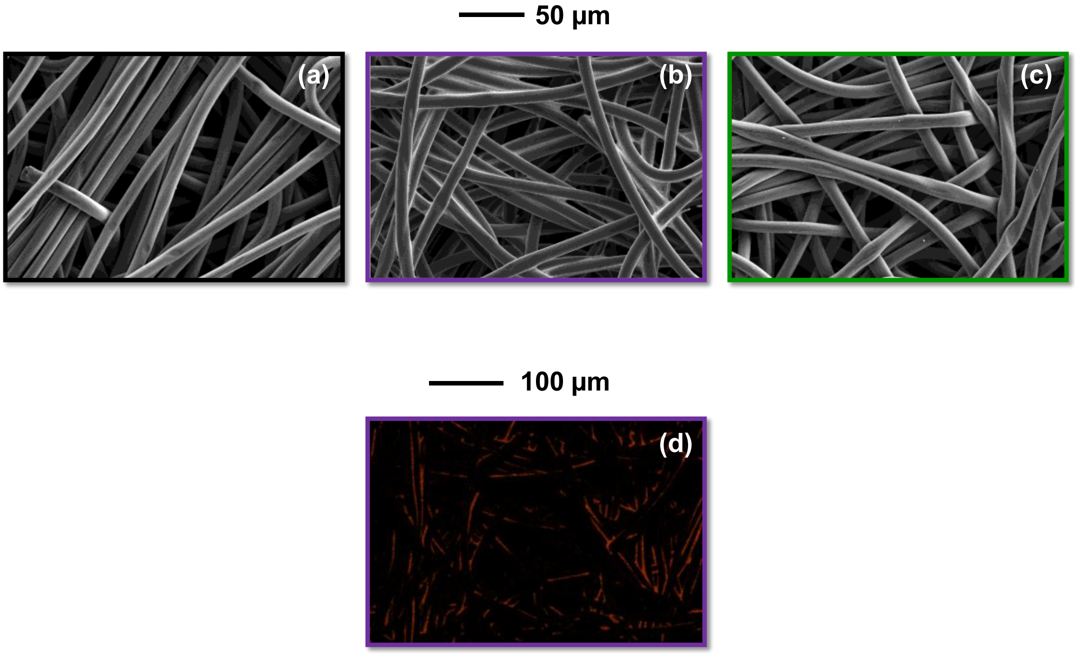

Figure S3. SEM images (accelerating voltage 10.0 kV, working distance 10 mm, probe current 45) of **(a)** untreated FH15, **(b)** PTFE dip-coated (10 wt. % loading) FH15 and **(c)** PDMS electrografted FH15; **(c)** EDX mapping of a PTFE dip-coated (10 wt. %) GDL, highlighting the uneven polymer distribution.

Cyclic voltammetry with redox probes

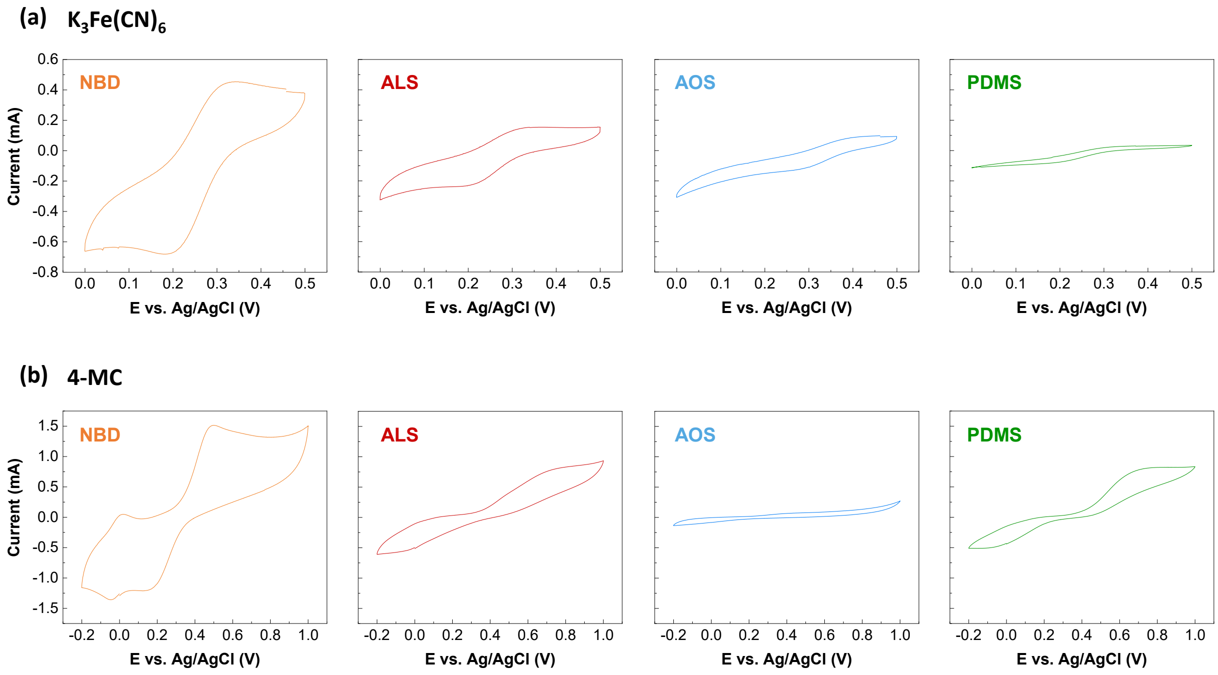

Figure S4. Zoomed-in voltammograms (last cycle of 5, scan rate 100 mV/s) of the electrografted GDLs, in aqueous electrolyte (KCl 1.0 M) with **(a)** ferricyanide (1 mM) and with **(b)** 4-methylcathecol (1 mM).

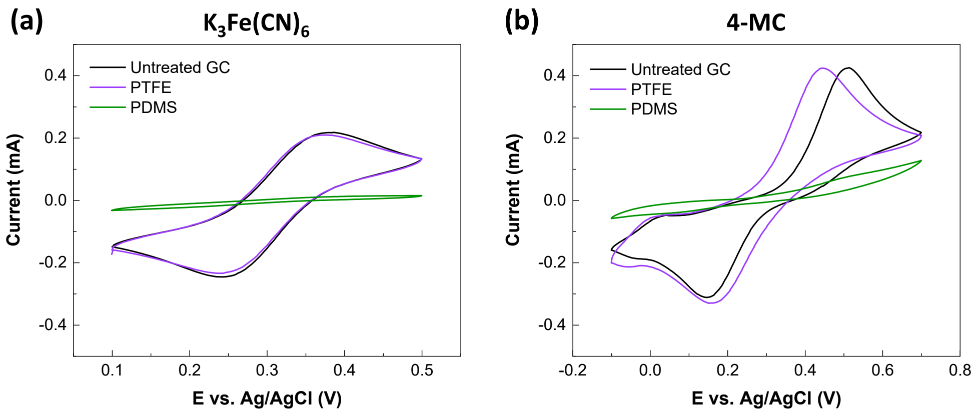

Figure S5. Cyclic voltammetry of ferricyanide (10 mM) on the left and 4-methylcathecol (10 mM) on the right in KCl 1.0 M, 5 cycles at 100 mV/s, on untreated GC, PTFE spin-coated GC and PDMS electrografted GC.

X-Ray Photoelectron Spectroscopy

Ar-Etching

The same etching experiments were carried out on the electrografted GDLs and on the PDMS electrografted GC. The experiment was performed only on one of the three electrografted GCs since it was observed that a single etching cycle was sufficient to remove the layer with the highest content of Si. This indicated that that the same results would likely apply to the other two samples.

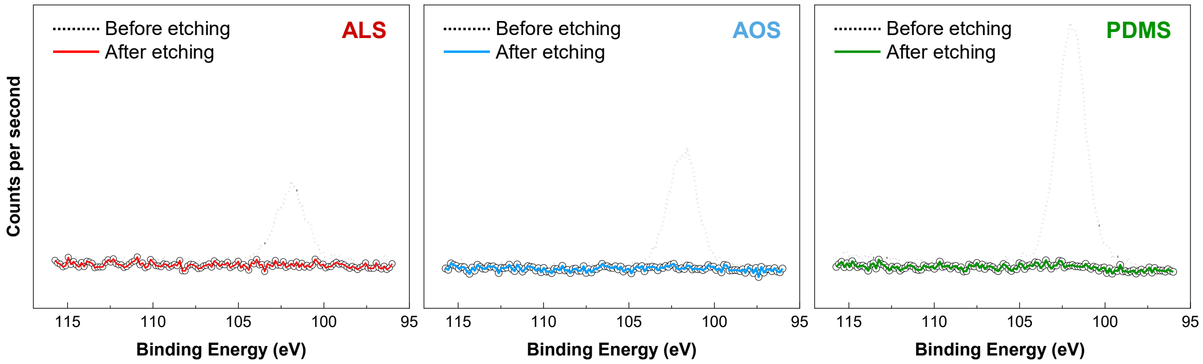

Figure S6. Si 2p peaks of the electrografted GDLs, before and after etching (60 etching cycles).

Contact angle and solid surface energy measurements

Table S1. Test fluids and their surface tension with dispersive and polar components.

SOLVENT	γ^{tot} (mN/m)	γ^{d} (mN/m)	γ^{p}(mN/m)
Methanol[1]	22.5	15.8	6.7
Ethanol[1]	23.2	21.2	2.0
N-Propanol[1]	23.9	22.1	1.8
Acetone[2]	26.0	19.0	7.0
Water[2]	72.8	21.8	51.0

Table S2. External contact angle values on the untreated GC, a PTFE sheet and the electrografted GCs with the different test liquids. Each one of the two tables relates to a different batch of samples, and three droplets were measured and averaged on each sample.

	GC		PTFE		NBD		ALS		AOS		PDMS	
	***θ* (°)**	**Error**	***θ* (°)**	**Error**	***θ* (°)**	**Error**	***θ* (°)**	**Error**	***θ* (°)**	**Error**	***θ* (°)**	**Error**
Methanol	12	± 2	36	± 2	10	± 3	0	± 0	0	± 0	0	± 0
Ethanol	10	± 1	30	± 5	8	± 6	7	± 0	15	± 2	16	± 2
N-Propanol	0	± 0	33	± 3	5	± 1	0	± 0	0	± 0	0	± 0
Acetone	11	± 1	33	± 2	12	± 2	14	± 1	18	± 1	20	± 1
Water	65	± 5	109	± 6	76	± 1	86	± 2	92	± 1	97	± 1

	GC		PTFE		NBD		ALS		AOS		PDMS	
	***θ* (°)**	**Error**	***θ* (°)**	**Error**	***θ* (°)**	**Error**	***θ* (°)**	**Error**	***θ* (°)**	**Error**	***θ* (°)**	**Error**
Methanol	0	± 0	42	± 2	5	± 1	15	± 1	14	± 3	16	± 3
Ethanol	0	± 0	29	± 2	5	± 3	0	± 0	0	± 0	0	± 0
N-Propanol	0	± 0	36	± 6	3	± 1	8	± 1	8	± 2	10	± 1
Acetone	0	± 0	32	± 3	9	± 2	10	± 1	10	± 1	16	± 2
Water	63	± 2	111	± 2	70	± 1	85	± 1	79	± 3	88	± 2

Table S3. External water contact angle values on the untreated gas diffusion layer substrate (FH15), the 10 wt. % PTFE dip-coated gas diffusion layer, and the electrografted gas diffusion layer. All droplets had a volume of 2 μL and an ellipse fitting was used. Each value is the average of three different measurements.

SAMPLE	Water CA (°)	Error
FH15	130	± 4
10% PTFE	150	± 2
NBD	75	± 8
ALS	132	± 3
AOS	132	± 6
PDMS	140	± 5

Table S4. External water contact angle values on the untreated GC, the PTFE sheet and the electrografted GC. All droplets had a volume of 5 μL and an ellipse fitting was used. Each value is the average of six different measurements (three measurements per sample from two different batches).

SAMPLE	Water CA	Error
GC	64	± 1
PTFE sheet	110	± 1
NBD	76	± 1
ALS	86	± 1
AOS	85	± 7
PDMS	92	± 5

Owens-Wendt method

The Owens-Wendt theory enables the calculation of the surface energy of solid materials. It relies on known surface tension values of specific test liquids and the measured contact angle of the solid surfaces. By combining the Good-Fowkes equation with the Young equation, it yield this linear relationship:

$$\underbrace{(1+cos\theta)\frac{\gamma_{LV}}{2\sqrt{\gamma_{LV}^{d}}}}_{y} = \overbrace{\sqrt{\gamma_{SV}^{d}}}^{q} + \underbrace{\sqrt{\frac{\gamma_{LV}^{p}}{\gamma_{LV}^{d}}}}_{x}\overbrace{\sqrt{\gamma_{SV}^{p}}}^{m}$$

where θ is the measured contact angle between liquid and solid, and γ_{LV}^{d} and γ_{LV}^{p} are known. The two unknown components of the solid surface energy, γ_{SV}^{d} and γ_{SV}^{p}, can then be determined by substituting the variables with the known values. A plot of **y** = $(1 + \cos\theta)(\gamma_{LV}/\ 2\sqrt{\gamma_{LV}^{d}})$ versus **x** = $\sqrt{\gamma_{LV}^{p}/\gamma_{LV}^{d}}$ for the different liquids gives the dispersive component γ_{SV}^{d} (square of the y-intercept, **q**), the polar component γ_{SV}^{p} (square of the slope, **m**) and consequently the surface tension of the solid–vapor interface γ_{SV}.

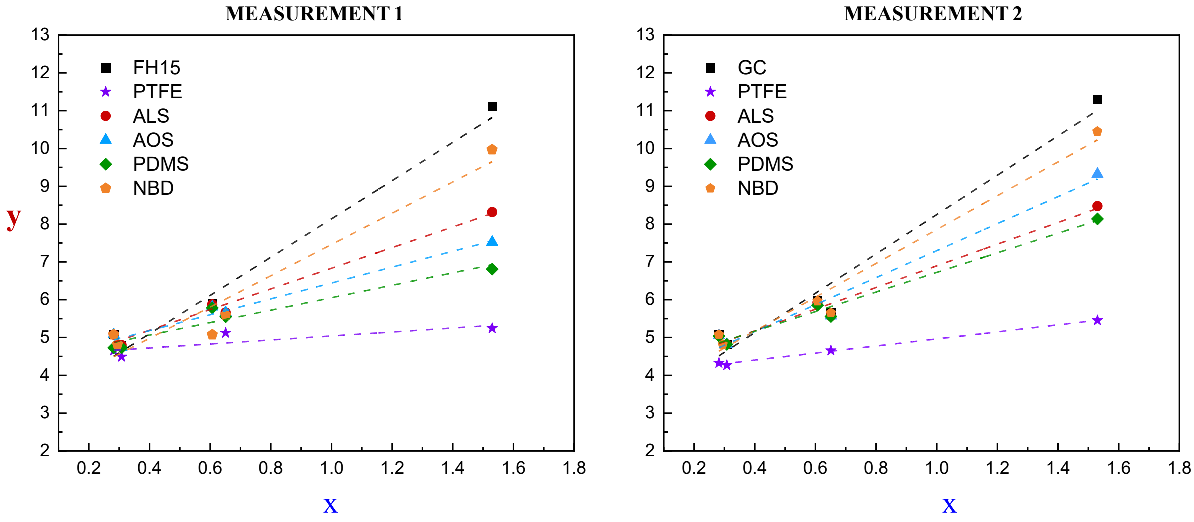

Figure S7. Owens-Wendt linear relationship for the untreated GC, the PTFE sheet and the electrografted GC.

Table S5. Values obtained from the linear extrapolation.

MEASUREMENT 1	m	q	R^2
GC	5.065	3.074	0.9605
PTFE	0.527	4.514	0.7182
NBD	3.856	3.618	0.9733
ALS	2.734	4.099	0.9838
AOS	2.100	4.343	0.9728
PDMS	1.512	4.567	0.9233

MEASUREMENT 2	m	q	R^2
GC	5.207	3.046	0.9623
PTFE	0.933	4.029	0.9954
NBD	4.818	3.221	0.9641
ALS	2.868	4.029	0.9780
AOS	3.575	3.723	0.9742
PDMS	2.594	4.128	0.9803

N.B. The solvents used for the linear fitting included methanol, ethanol, n-propanol, acetone and water, except for PTFE, where acetone had to be excluded. Unlike alcohols, which have hydroxyl groups that may interact in a more predictable way with PTFE, the interactions with acetone, which has a polar carbonyl group, might be inconsistent, disrupting the expected trend in surface energy calculations. Moreover, the significant difference in acetone contact angle can disrupt the linearity leading to inaccuracies in surface energy estimations.

Single-cell testing

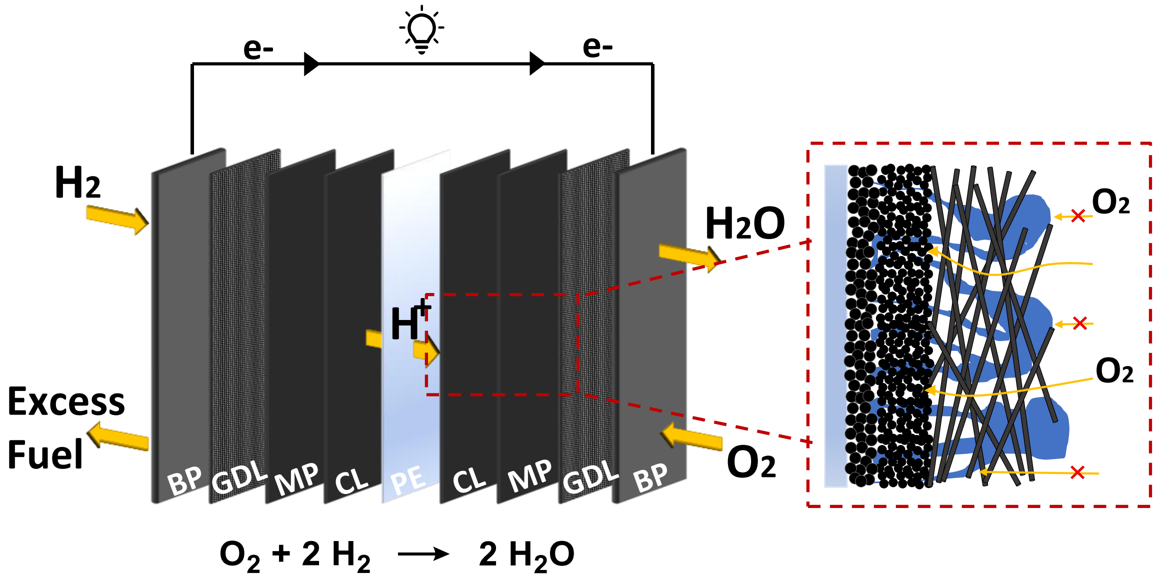

Figure S8. Illustration of a PEFC and of the water issue on the gas diffusion media.

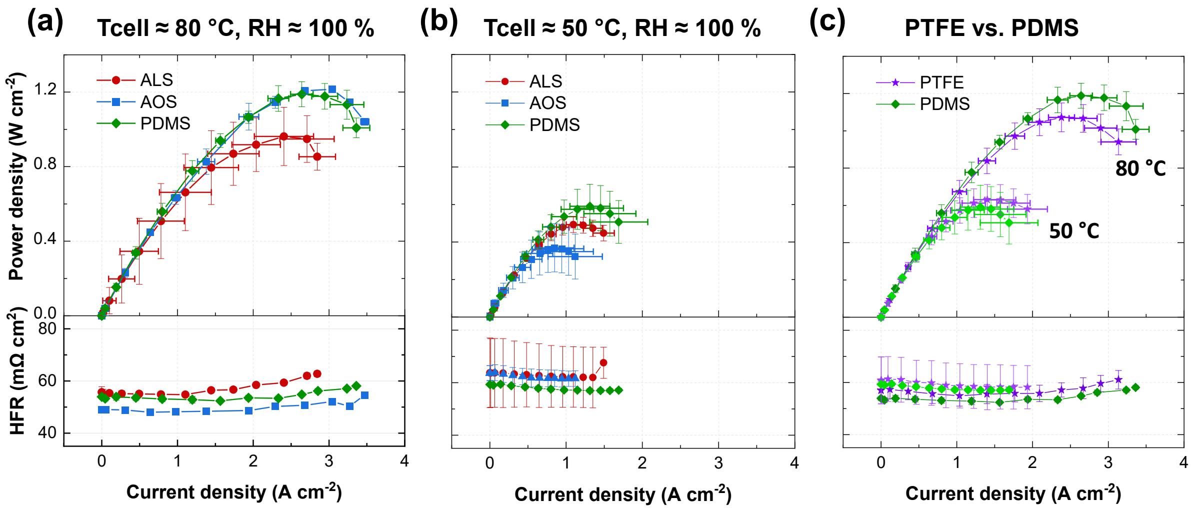

Figure S9. *Differential-flow H_2/Air (0.5/5 nlpm) polarization curves showing power density (top) and high frequency resistance (bottom) vs. current density for the cathode electrografted GDLs and for the state-of-the art GDL.* Operating conditions are: **(a)** T_{cell} = 80 °C, RH = 100 %, p_{abs} = 150 kPa; **b)** T_{cell} = 50 °C, RH = 100 %, p_{abs} = 150 kPa; **c)** T_{cell} = 50 °C vs. T_{cell} = 80 °C, RH = 100 %, p_{abs} = 150 kPa. The error bars represent the standard deviation of three independent measurements.

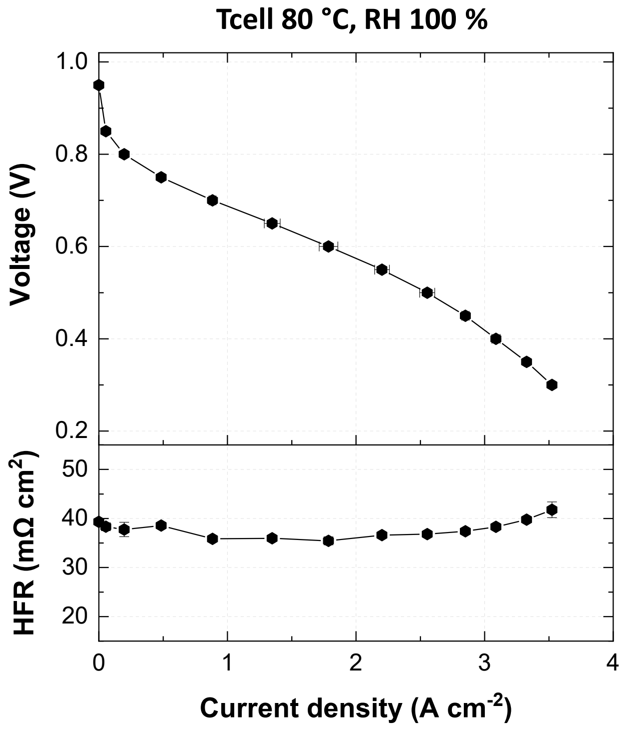

Figure S10. *Differential-flow H_2/Air (0.5/5 nlpm) polarization curve showing cell voltage (top) and high frequency resistance (bottom) vs. current density for commercial GDL+MPL (Freudenberg H15C14) at cathode and anode.* Operating conditions are Tcell = 80 °C, RH = 100 %, p_{abs} = 150 kPa. The error bars represent the standard deviation of two independent measurements.

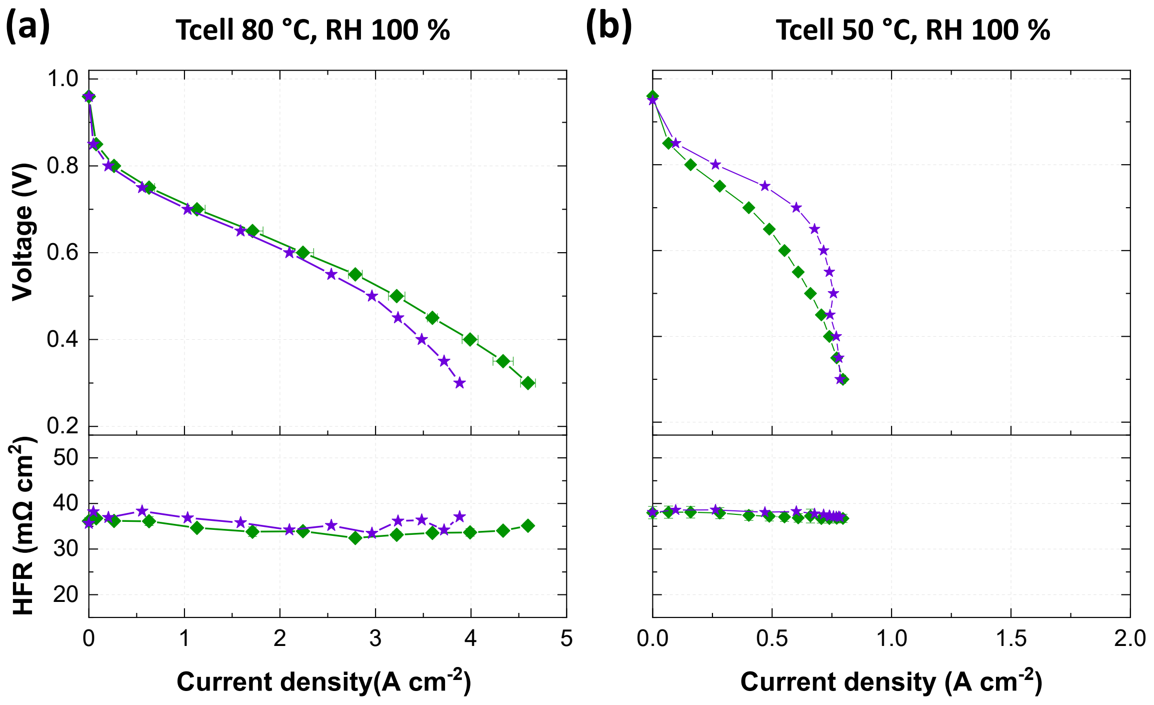

Figure S11. *Differential-flow H_2/Air (0.5/5 nlpm) polarization curves showing cell voltage (top) and high frequency resistance (bottom) vs. current density for the cathode electrografted GDLs and for the state-of-the art GDL (**Toray TGP H060**).* Operating conditions are: (**a**) Tcell = 80 °C, RH = 100 %, p_{abs} = 150 kPa; (**b**) Tcell = 50 °C, RH = 100 %, p_{abs} = 150 kPa. The error bars represent the standard deviation of two independent measurements.

References

(1) Parry, V.; Appert, E.; Joud, J.-C. Characterisation of Wettability in Gas Diffusion Layer in Proton Exchange Membrane Fuel Cells. *Applied Surface Science* **2010**, *256* (8), 2474–2478.

(2) Zdziennicka, A.; Krawczyk, J.; Szymczyk, K.; Jańczuk, B. Components and Parameters of Liquids and Some Polymers Surface Tension at Different Temperature. *Colloids and Surfaces A: Physicochemical and Engineering Aspects* **2017**, *529*, 864–875.
